# An evaluation of the provision of oncology rehabilitation services via telemedicine using a participatory design approach

**DOI:** 10.1007/s00520-021-06552-8

**Published:** 2021-09-23

**Authors:** Grainne Brady, Kate Ashforth, Siobhan Cowan-Dickie, Sarah Dewhurst, Natalie Harris, Alline Monteiro, Catherine Sandsund, Justin Roe

**Affiliations:** 1 Therapies Department, The Royal Marsden NHS Foundation Trust, London, UK; 2 Department of Surgery & Cancer, Imperial College London, London, UK; 3 Department of Otolaryngology, Head and Neck Surgery, Imperial College Healthcare NHS Trust, London, UK

**Keywords:** Rehabilitation, Telemedicine, COVID-19, Oncology, Service delivery, Experience-based co-design

## Abstract

**Background:**

The COVID-19 pandemic has fundamentally impacted the delivery of healthcare services globally. In line with UK government guidelines on social distancing, the use of telemedicine was implemented to facilitate the ongoing provision of cancer rehabilitation.

**Purpose:**

We sought to evaluate and co-design telemedicine services to meet the complex needs of our patients and carers at a tertiary cancer centre.

**Methods:**

Experience-based co-design methodology was adapted to include virtual methods. Staff members (*n* = 12) and patients (*n* = 11) who had delivered or received therapies services at our UK cancer centre since March 2020 were recruited to take part in one-to-one virtual interviews. Patient interviews were video recorded, analysed and edited to a 30-min “trigger film”. Patient and staff virtual events were undertaken thereafter. A joint virtual patient and staff event occurred. Staff and patients watched the trigger film and as partners, agreed areas for change and developed groups for service co-design.

**Results:**

Positive aspects regarding telemedicine provision were highlighted including reduced financial and time burden on patients, and increased flexibility for both staff and patients. The key concerns included digital exclusion, safety, communication and patient choice. Four co-design groups have been established to enact changes in these priority areas.

**Conclusion:**

Using a participatory design approach, we have worked in partnership with patients and staff to ensure the safe, acceptable and effective delivery of rehabilitation services with integrated telemedicine.

## Introduction

The majority of patients living with and beyond cancer will experience physical, cognitive and emotional impacts as a result of their cancer diagnosis and/or treatments received [1]. Functional impairments before and following a cancer diagnosis and subsequent treatment can impact an individual’s social and vocational roles and can result in poorer survival outcomes [2]. Rehabilitation services provide vital interventions for people aiming to reduce the potential impact of cancer and cancer treatments on physical, social, emotional and cognitive functioning [2].

The COVID-19 pandemic has fundamentally impacted the delivery of cancer services globally and in response there was a rapid reorganisation of services to ensure that patients continued to receive essential care while minimizing exposure to the virus [3]. Cancer rehabilitation services at our UK-based centre also had to change quickly to comply with government guidelines on social distancing, to reduce footfall across our tertiary cancer referral centre and to help reduce potential transmission to our patients, many of whom were classified as ‘clinically extremely vulnerable’ patients [4]. Changes included the implementation of telemedicine services including video and telephone consultations, to ensure the ongoing provision of rehabilitation support and interventions.

‘Telemedicine’ and ‘telehealth’ are terms which are sometimes used interchangeably however there are two separate definitions in the literature. Telemedicine is described as the use of communication technology in healthcare in which the clinician and patient involved are at different locations during the consultation [5]. Telehealth involves the remote exchange of data between a patient and healthcare professionals as part of the patient’s diagnosis and healthcare management [6]. For the purposes of this paper, the term telemedicine is used to describe the use of telephone or video consultations for the provision of rehabilitation services to patients.

Telephone consultations have been used in primary care for over 100 years and the first documented use of video consultations occurred in 1964 in the oncology setting where patients accessed opinions on skin lesions from a melanoma consultant based at a different hospital [7]. With regards to oncology rehabilitation services, the use of telemedicine has long pre-dated this global pandemic, in particular for the ongoing care of oncology patients based in remote areas in Australia [8, 9]. Although the benefits of telemedicine for patients living with and beyond cancer in the UK including independence and reassurance have previously been reported in the literature [10], telemedicine was not widely used in the routine care of oncology patients within the UK National Health Service (NHS) prior to the pandemic [11].

Acknowledging the balance between missed care opportunities and COVID-19 transmission risks to both patients and health care providers during the pandemic, recommendations for practical approaches to managing patients with cancer during the pandemic were made [12]. Based on experience delivering cancer care during the SARS epidemic, telemedicine was advocated as a possibility for delivering some aspects of outpatient supportive care, with evidence suggesting that it improves access to care and reduces healthcare costs [12].

The COVID-19 pandemic has caused significant human, financial and social costs to the UK and beyond. There have been some positives, in particular the innovation and flexibility seen within the UK National Health Service and the short timescales where rapid change has occurred. Certain changes in healthcare service provision may remain on a long-term basis including the more widespread use of telemedicine. At a local level, although the telemedicine delivery was acceptable and effective for some patients, digital exclusion remained a significant problem. Concern regarding the accessibility to and acceptability of such services for all, and staff apprehension in delivering the majority of services in a non- face-to-face manner, necessitated the need to evaluate the rapid service development during the pandemic. Service evaluation and quality improvement (QI) are likely to be critical processes to facilitate the successful provision of new models of service delivery post-pandemic. QI and service evaluation can involve various methodologies to seek feedback that are then used to design or redesign services [13]. Previous quantitative studies have examined patient characteristics associated with choosing a telemedicine visit vs face-to-face consultation [14] and studies have used survey/questionnaire design to assess patient and healthcare providers’ views on the use of telemedicine services [11, 15]. Such quantitative methods may not capture the essence of patient and healthcare provider experience [16]. In recent years there has been an increased focus in the literature on moving towards co-design methods of service design [17]. Rather than using questionnaires to seek feedback on suggested changes in health care processes and services, co-design is a joint venture that involves service users and health care professionals working together as the co-designers of a service [18].

At our tertiary cancer referral service, the rehabilitation team comprises of a range of allied health professionals including Dietitians (DT), Occupational Therapists (OT), Physiotherapists (PT), Lymphoedema Therapists (LT) and Speech and Language Therapists (SLT). The rehabilitation team works across a range of tumour groups for example breast, gastrointestinal, head and neck/thyroid, neuro-oncology, urology and gynaecology and have a critical role in supporting and rehabilitating people affected by cancer with highly specialist knowledge and skills. People living with cancer are a heterogeneous population, embarking on differing care pathways with varying survival and recovery needs. The rehabilitation team provides input prior to treatment (prehabilitation), support during treatment and ongoing rehabilitation to those patients living with and beyond cancer treatment. Before the pandemic, our services were delivered in an almost exclusive face-to-face manner. Given the rapid implementation of telemedicine to continue to support the rehabilitative needs of our patients during the pandemic, we sought to evaluate the provision of oncology rehabilitation services via telemedicine using experience based co-design methodology (EBCD) [19].

## Methodology

**Fig. 1:**
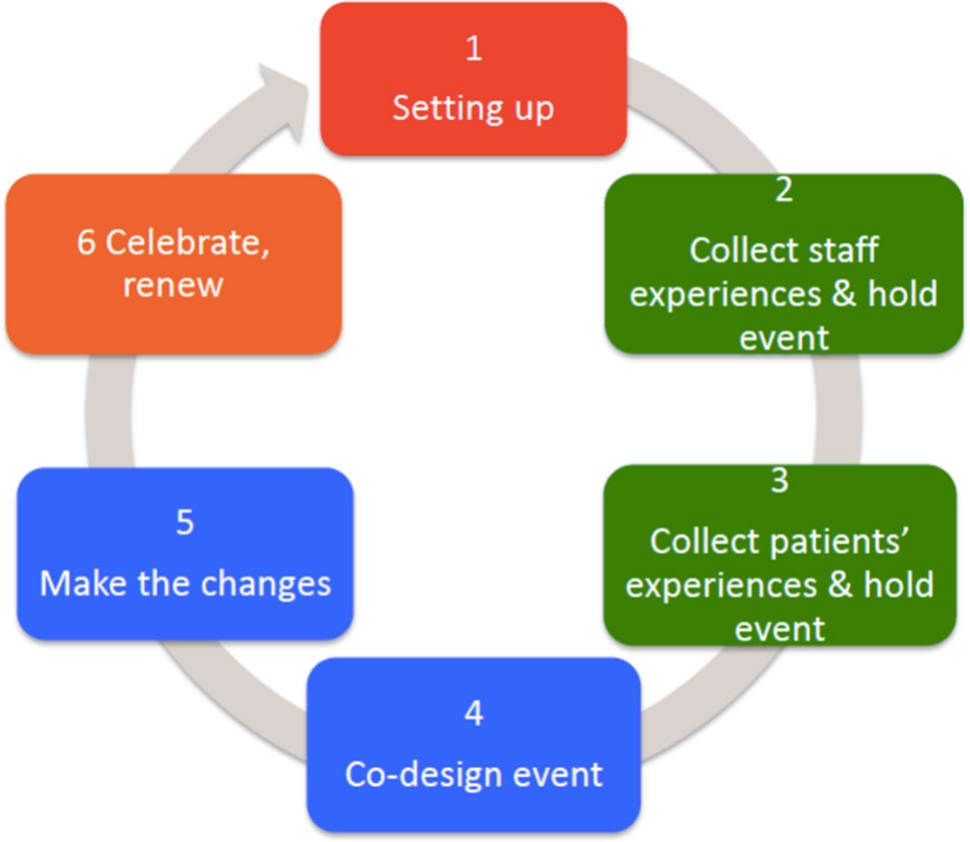
EBCD methodology [19]

Experienced-based co-design (EBCD) is an approach that enables staff and patients to co-design services in partnership using six key stages (Fig. 1).

The project was approved by the Royal Marsden NHS Foundation Trust’s Committee for Clinical Research (SE961) in July 2020. Patients and staff who met the eligibility criteria were invited to take part in in-depth semi structured interviews exploring their individual experiences of telemedicine rehabilitation services at the hospital since March 2020. In line with EBCD methodology [19]; the target sample size was approximately 12 staff and 12 patient participants. Patients who were not comfortable in using/appearing in online meeting were also invited to contribute via written narrative. Staff and patient inclusion/exclusion criteria are summarised in Table 1.

**Table 1:** Inclusion/exclusion criteria

Inclusion criteria (patients):	Inclusion criteria (staff):
1. 18 years of age or over 2. adequate linguistic and cognitive function to participate in interviews and/or group discussions 3. have been offered and/or taken part in telemedicine rehabilitation services during the COVID-19 pandemic	1. staff who have undertaken rehabilitative interventions using telemedicine services or supported others to deliver them during the COVID-19 pandemic
Exclusion criteria (patients):	Exclusion criteria (staff):
Does not meet all of the inclusion criteria	Does not meet all of the inclusion criteria

Interviews were audio recorded (staff) and video recorded (patients) and were transcribed and main themes identified. Patient interviews were compiled into a 30-min “trigger film” to summarise the key themes from the patient interviews. Staff members were then invited to a staff group discussion to identify priorities for change in service provision whilst patients were invited to a patient group discussion where they could view the edited video. The video was used to stimulate a patient group discussion where key ‘emotional touch points’ (emotionally significant points) were identified and either positive or negatives feelings were assigned based on individual experience. The staff and patient participants then attended a joint event. The trigger film was shown to both staff and patients, conveying to staff how patients experienced telemedicine services. Further group discussion was undertaken to identify joint staff and patient priority areas for change and smaller co-design groups were established to work on these areas.

## Results

Eleven patients and 12 staff members were recruited to take part in the study. No written narratives were submitted. Patient and staff demographics are summarised in Table 2.

**Table 2:** Patient and staff demographics

Patients (*n* = 11)	Staff (*n* = 12)
Male: 5 Female: 6	Male: 1 Female: 11
Mean age (range): 59 (37–77)	Professional role: Divisional lead: 1 Therapies lead: 1 Speech & language therapist: 2 Physiotherapist: 1 Lymphoedema therapist: 2 Exercise specialist: 1 Dietitian: 2 Administrator: 2
Tumour type: Head & neck/thyroid: 7 Gastrointestinal: 1 Breast: 2 Haematology: 1	

All the patients had engaged in a total of 80 telemedicine consultations prior to being interviewed with a median number of telemedicine consults of 7 per patient (range 2–15). This included experience of both telephone consultations and video consultations. A total number of 24 telephone consultations was undertaken, and 56 video consultations. The majority of consultations (*n* = 39) involved uni-disciplinary video consultations including 20 SLT video calls, 12 PT video calls and 7 LT video calls. Multidisciplinary video consultations (*n* = 15) included both joint DT and PT pre-surgical assessment and advice and joint SLT and DT pre-head & neck/thyroid assessment and advice. Joint telephone DT and SLT telephone consultations (*n* = 9) were also undertaken for the purposes of on-treatment monitoring and advice for head & neck/thyroid patients undergoing radiation treatment. The majority of consultations (*n* = 61) were focused on rehabilitation after treatment across head neck & thyroid, breast, gastrointestinal, and urology tumour groups. The nature/type of telemedicine consultations are summarised in Table 3.

**Table 3:** Telemedicine consultations

Telemedicine consultations	Telephone: 24 Video: 56		
Uni-disciplinary:	Telephone	Video	
	DT: 9	DT: 0	
	SLT: 2 PT: 4	SLT: 20 PT: 12	
	LT: 2	LT: 7	
Multi-disciplinary:	Telephone	Video	
	SLT + DT: 9	PT + DT: 1 SLT + DT:15	
	*n*	Focus of consultations:	
Pre-treatment	3	Joint PT and DT breast cancer pre-surgical assessment & optimisation (prehabilitation) Joint SLT and DT head & neck/thyroid pre-radiation assessment & optimisation (prehabilitation) Joint SLT and DT head & neck/thyroid radiation on-treatment review SLT post head & neck radiation swallowing rehabilitation Joint SLT/DT post head & neck/thyroid radiation nutrition and swallowing review Post cancer treatment PT exercise rehabilitation (individual) Post cancer treatment PT led exercise rehabilitation (group classes) Post gastrointestinal cancer treatment dietetic review	
On-treatment	16		
F/u post treatment	61		

Eight key themes were identified across staff and patient interviews which are summarised in Table 4. These included cost effectiveness and efficiency, flexibility, patient centred care, importance of face-to-face consultations, safety, change, training, and inclusivity.

**Table 4:** Interview themes

Themes	Staff	Patients
Cost effectiveness & efficiency	‘Reduced hospital transport costs…no longer having to pay for hospital transport for our patients’ ‘reduced delays in clinic with patients arriving late due to transport issues’	‘No travel- ongoing care despite living many miles from hospital’ ‘Increased access to therapy support’ saves travel, money and less disruption when working’
Flexibility	‘More flexible with how we work, because of working from home I changed my hours. I condensed my four days in three and I am doing three long days’	‘much easier for the patient’ ‘questions answered from the comfort of home’ ‘one hour off work rather than a day/half a day’
Patient centred	‘there are advantages to seeing them in their home environment, sometimes it is quite nice, because seeing them in a hospital you don’t really get a sense of what their set up at home is and their support’ ‘It is nice to see them in their home, where they are comfortable and family members can join’	‘much easier for patient to be at home’ ‘the answers to the questions, no travel required and no mask’
Importance of face-to-face	‘If you can’t do face to face, the next best thing is the video and then lastly telephone’ ‘I think having that [video], building up a rapport is so much easier than just on the phone. I found it very helpful when you are still getting to know patients in a little bit’ ‘unable to do a physical exam’	‘Face to face interactions are better if something has to be shown/seen’ ‘Videos not good for physical examination’ ‘Meeting old friends- virtual a bit more impersonal’ ‘Telephone not appropriate when more than one professional there’ ‘Phone calls are less intrusive than video consults. Coming to the hospital helps prepare for the consult/review’
Safety	‘We had to be a bit careful in terms of the safety aspects of doing these virtual sessions. Obviously, you are not there to catch someone if they fall and you cannot measure someone's heart rate.’ ‘You cannot really see how they are working… that is something which we have had to write into protocols’	‘element of guess work in absence of face to face, is professional getting the full story?’
Change	‘We went from a service with no telehealth to a service that was 100% telehealth’ ‘The speed of the transition was very impressive for the NHS. I think one positive has been that now we can implement change much quicker than ever we would have considered’	‘before March 2020, nobody knew what zoom was’
Use of technology	‘clinicians didn't have a dedicated space to contact patients’ ‘a lot of our clinic rooms do not have IT access or telephone access. Also, our team sit in shared offices, which are not suitable to have consultations with patients’ ‘There are some general technical issues, like the signal is poor at home, the screen is freezing and not being able to hear me…. It can be disruptive in an appointment and a bit frustrating… if they can't hear what I am saying, or we are just freezing every few minutes… it just ends up taking quite a long time. ‘	‘able to initiate own access to appointment by email. Received detailed guidance, felt safe and privacy was respected’ ‘I was OK but my mum wouldn’t be able to do it. It’s good for my generation’ ‘Technology may cause others to panic’
Training	‘needs to be training on how to deliver a video consultation, in terms of, when you are doing during the consultation, the posture, the language that you use, hand movements, how you talk, what information you share, where you sit, where is your camera, all of those things’ ‘Patients also require training’ ‘Something to take them step by step though how to go onto [the call]. ‘Even if they [patients] have instructions, sometimes they struggle to follow that and I think that delays clinic.’	‘received appt via email with follow up text also containing tech support which was very helpful’ ‘use video conferencing for work every day’ ‘other patients who are older might feel panicked’
Inclusivity	‘communication difficulties’ ‘language barriers can make telemedicine very difficult’	‘Older patients may struggle’

Staff and patient participants recognised the cost effectiveness of remote consultations. Patients highlighted how they did not incur travel costs with reduced time burden. Flexibility was also noted by patients for example, if taking time off work, they could take the time designated for the appointment rather than a number of hours to a day off to attend a hospital based appointment. Staff also noted that they could work more flexibly with remote clinics, adjusting working schedules to complete remote clinics from their home. Staff and patients also highlighted the unique opportunity to see people in their homes where they presumably feel most comfortable. It also allowed family members to join the consultation if and when appropriate. All participants noted that there needs to be a balance between telemedicine consultations and face-to-face consultations highlighting the need post-pandemic to have a flexible and responsive approach. Video consultations rather than telephone consultations were favoured by all, particularly in the multidisciplinary setting. It was highlighted that when a physical examination of the patient is required, that a face-to-face consultation would be needed. This links closely with the safety theme where staff and patients highlighted concerns regarding any potential risk to patients for example a fall during an exercise class or if a symptom/observation is missed due to a lack of physical examination. The pace of change to embed telemedicine was celebrated by both staff and patients and although patients felt confident regarding training requirements and use of technology, staff highlighted the need for specific training to deliver telemedicine consultations. The need for appropriate infrastructure was raised, including private spaces for consultations, a reliable and safe platform for patient consultations including stable internet access. All patient and staff participants were able to engage in telemedicine consultations. Concerns were raised regarding those patients who may be digitally excluded for a variety of reasons including speech, voice, language or cognitive difficulties or indeed if patients did not have the devices and knowledge.

The key themes of the staff and patient interviews are summarised in Table 4.

Based on the 8 key interview themes, patients and staff members identified four key co-design groups including:

1. *Inclusivity* focusing on developing a telemedicine service which is accessible to all, limiting and preventing any potential digital exclusion.
2. *Safety* ensuring our interventions provided via telemedicine are safe
3. *Communication* enhancing communication to patients regarding the range and choice of telemedicine and face-to-face appointments available and how patients may access these
4. *Peer-training* the use of peer training to enhance patient access to telemedicine services

While the four co-design groups worked on their individual areas, it was noted that there was a lot of overlap between the four co-design areas. Collectively the following key areas for change were agreed:

1. ‘Patient first not digital first’
  Unlike during the height of the pandemic where the default appointment offered was a telemedicine consultation- post pandemic it was felt that the choice of appointment type should be patient centred. It was suggested that in some circumstances that a telemedicine consultation may focus a subsequent face-to-face consultation if specific examinations, assessments or interventions would be required. However, it is important to clearly communicate to each patient the choices/rationale for the different types of appointments available. It must also be highlighted that there is flexibility between telemedicine and face-to-face based on patient needs and wishes.
2. ‘Safe service’
  There is a need to develop a standard operating procedure to ensure that each telemedicine consultation is conducted in a safe manner to avoid any potential risk to patients. This will include an assessment of the need for physical examination.
3. ‘Clear communication’
  Patients should be able to register their preferences for communication (letter, email, telephone) at the point of registration and indicate their availability/willingness to engage in telemedicine consultations
4. ‘Inclusive service’

Telemedicine should be an option for all patients if they wish to partake, all efforts should be made to ensure that patients have the access to technology and knowledge to use it to facilitate engagement in telemedicine consultations.

## Discussion

We used EBCD to evaluate and start co-designing oncology rehabilitation telemedicine services at a tertiary cancer centre. Staff and patient experience data was used to gain an in-depth understanding of the positive aspects of telemedicine and the areas for further development and refinement. We continue to work with our patient partners to co-design an oncology rehabilitation service with integrated telemedicine which is available to all, if and when appropriate and safe to use.

In line with previous research the positives of telemedicine were highlighted by both patients and staff members, [8, 12] however, our patients and staff had a strong preference for a mix of both face-to-face and telemedicine to provide the highest quality standard of care. Contrary to recent research [12], both healthcare professionals and patients highlighted their concerns regarding the lack of physical examination and that in certain circumstances a direct physical examination would be required to provide safe and effective care. This would necessitate clear safety protocols to identify if and when a telemedicine consult would be appropriate and also clear methods of communication to the patient so that they can express their preference. At our centre we are currently co-designing a standard operating procedure for telemedicine consultation to ensure our service is both safe and person centred.

We now offer a hybrid approach with a mixture of face-to-face and telemedicine consultations. Some examples of this include a newly designed remote swallowing bootcamp intervention for head and neck patients delivered by the SLT department. Here the patients need to be seen for face-to-face for assessment of eligibility including an instrumental evaluation of swallowing. Once all eligibility assessments have been completed the patient is enrolled into a daily therapy session delivered via video consultation. The SLT administrator speaks to the patient via telephone to ensure they have the suitable equipment and are able to join the consultation. Likewise, although face-to-face individual physiotherapy musculoskeletal assessments continued in pandemic with strict criteria, the initial assessment is now either virtual or face to face, depending on patient choice and clinical need, including for physical examination. As part of the prehabilitation process for those diagnosed with upper gastrointestinal cancers, physiotherapy assessment is being carried out face to face in clinic. Group rehabilitation exercise classes continue to be delivered online. We try to avoid multidisciplinary telephone calls to patients offering video calls as standard. We continue to work with our IT department to ensure that we have a secure and reliable platform for video consultations. Each member of the rehabilitation team now has an audio headset to improve the quality of the sound and privacy during consultations.

Both patients and professionals found telemedicine to be efficient as in previous studies [8, 12] however, this was only the case when the technology performed as it should. Our study has highlighted that if there are IT issues, or indeed if the patient does not fully understand the instructions for joining a virtual consultation that this can reduce the efficiency of a clinic. This highlights the need to enhance patient communication/training regarding access to telemedicine platforms. At our centre we continue to try to provide patients with as much information as possible to try and make joining a telemedicine consultation as smooth and stress free for the patient as possible. We continue to co-design peer training pathways to reduce digital exclusion and are looking to engage in charitable partnerships to ensure that patients have access to the required technology to engage in telemedicine consultations.

In line with previous research, we have found that successful telemedicine provision is more than just a question of technology. It requires fundamental changes in service design, with collaboration as a key determinant in successful implementation [20]. We sought to achieve this through collaboration and co-design with our patient partners and healthcare providers.

## Conclusion

We have worked in partnership with patients and staff to ensure that we can deliver telemedicine rehabilitation services in a co-designed and co-produced format. Through the use of EBCD, rich insights have been gained into the barriers and facilitators to a positive and effective patient and staff experience of telemedicine in the context of oncology rehabilitation. This ongoing project will ensure our service is accessible and meets our patients’ individual and varied needs. This project is not without its limitations including a key area of concern that patients who were unable to or did not wish to access telemedicine services were not included in the project. As the project was undertaken during the pandemic, all interviews, group discussion and events took place virtually so there was a selection bias in terms of our sample. As we continue with this work, and with the easing of restrictions, we hope to involve more patients with the project including understanding why and how telemedicine was not accessible or acceptable to them during the pandemic.

## Data Availability

Anonymised data available on request from the authors.

## References

[1] Miller KD, Siegel RL, Lin CC, Mariotto AB, Kramer JL, Rowland JH, Stein KD, Alteri R, Jemal A (2016). Cancer treatment and survivorship statistics. CA Cancer J Clin.

[2] Stout NL, Santa Mina D, Lyons KD, Robb K, Silver JK (2021). A systematic review of rehabilitation and exercise recommendations in oncology guidelines. CA Cancer J Clin.

[3] Richards M, Anderson M, Carter P, Ebert BL, Mossialos E (2020). The impact of the COVID-19 pandemic on cancer care. Nat Cancer.

[4] Department of Health and Social Care 2021 Guidance on shielding and protecting extremely vulnerable persons from COVID 19. https://www.gov.uk/government/publications/guidance-on-shielding-and-protecting-extremely-vulnerable-persons-from-covid-19/. Accessed 1st June 2021

[5] Fitch CJ (1999). Telemedicine to support the elderly in the UK. Health Inform J.

[6] Steventon A (2012). Effect of telehealth on use of secondary care and mortality: findings from the Whole System Demonstrator cluster randomised trial. Br Med J.

[7] Stokel-Walker C (2020). Why telemedicine is here to stay. BMJ.

[8] Burns CL, Ward EC, Hill AJ, Kularatna S, Byrnes J, Kenny LM (2017). Randomized controlled trial of a multisite speech pathology telepractice service providing swallowing and communication intervention to patients with head and neck cancer: evaluation of service outcomes. Head Neck.

[9] Sabesan S, Brennan S (2011) Tele oncology for cancer care in rural Australia. Telemedicine techniques and applications, pp 289–306

[10] Cox A, Lucas G, Marcu A, Piano M, Grosvenor W, Mold F, Maguire R, Ream E (2017). Cancer survivors’ experience with telehealth: a systematic review and thematic synthesis. J Med Internet Res.

[11] Smrke A, Younger E, Wilson R, Husson O, Farag S, Merry E, Macklin-Doherty A, Cojocaru E, Arthur A, Benson C, Miah AB (2020). Telemedicine during the COVID-19 pandemic: impact on care for rare cancers. JCO Glob Oncol.

[12] Al-Shamsi HO (2020). A practical approach to the management of cancer patients during the novel Coronavirus Disease 2019 (COVID-19) pandemic: an international collaborative group. Oncologist.

[13] Brady GC, Roe JWG (2020). Whose service is it anyway? Patients as co-designers to improve dysphagia care pathways. SiG 13 Perspect Swallow Swallow Disord.

[14] Reed ME, Huang J, Graetz I, Lee C, Muelly E, Kennedy C, Kim E (2020). Patient characteristics associated with choosing a telemedicine visit vs office visit with the same primary care clinicians. JAMA Netw Open.

[15] Elawady A, Khalil A, Assaf O, Toure S, Cassidy C (2020) Telemedicine during COVID-19: a survey of health care professionals’ perceptions. Monaldi Arch Chest Dis 90(4) 10.4081/monaldi.2020.1528 32959627

[16] Brady GC, Goodrich J, Roe JW (2020). Using experience-based co-design to improve the pre-treatment care pathway for people diagnosed with head and neck cancer. Support Care Cancer.

[17] Robert G (2015). Patients and staff as codesigners of healthcare services. BMJ.

[18] Bate P, Robert G (2006). Experience-based design: from redesigning the system around the patient to co-designing services with the patient. Br Med J.

[19] The Point of care foundation (2021) Experience based co-design toolkit. https://www.pointofcarefoundation.org.uk/resource/experience-based-co-design-ebcd-toolkit/. Accessed 1st June 2021

[20] The Kings Fund (2021) The impact of telehealth: a review of the evidence*.* https://www.kingsfund.org.uk/projects/whole-systems-demonstrator-action-network-wsdan/impact-telehealth-review-evidence. Accessed 1st June 2021

